# Supplementary material for: Emergence of a smooth interface from growth of a dendritic network against a mechanosensitive contractile material
Source: eLife. 2021 Aug 23;10:e66929. doi: 10.7554/eLife.66929 (PMC8410080; doi:10.7554/eLife.66929)
Supplement: Source code 1. — MATLAB code is available at: https://github.com/medha7575/sharma-et-al-ELIFE2021. [file elife-66929-code1.zip › SharmaetalAnnotatedcodeforActomyosinAloneModel.pdf]

This file initiates the nodes of actomyosin material.

Table of Contents

It first defines the parameters and then generates both the passive and active nodes in a random fashion. 1
Thereafter it connects the nodes with neighors to create a connected network. 1
Following are the variables for easily assigning to array while initializing 1
Following are the parameters used 2
----- Myosin Initialization------------ 2
% ------making Node IDs-------- 4
-----Making Node neighbors---------- 5
Following lines calculate the mean resting length of connections 7
print an image of initial setup 7

It first defines the parameters and then generates both the passive and active nodes in a random fashion.

Thereafter it connects the nodes with neighors to create a connected network.

```
clc
clear all
close all


cellCenters=[250,250];%
```

Following are the variables for easily assigning to array while initializing

```
TOTAL_NODES_AT_LOCATION = 3;
NODE_ID_AT_LOCATION=4;

ROW=1;
COL=2;
MYO_CONC=3;
NO_OF_NEIGHBOR=4;
MOVEORNOT=5;
COMBOVALUE=6;
MEAN_LENOFCONNEC=7;
```

Following are the parameters used

```
% Lattice defination
bound=500;
rows=bound;
cols=bound;

Combo = zeros(bound,bound);% Used for visualizing the lattice

Myo=zeros(bound,bound);  % Used for visualizing the myosin activity
 field
Mc_NodeNo = ones(rows,cols).*-1; % Main array holding myosin material
 features


ActM=40;% Myosin activation region
MyoR=140;% Total area of myosin

Mc= 350 ; % Active myosin
Dm= 50; % Passive myosin

Den_m=0.3; % myosin density



% Other actomyosin material parameters
Myo_conc=0.9; %Myosin activity per node/concnetration ('M'in paper)
Dis_Thres_Neigh1=5;%500nm distance Myosin Connection Search Distance
 ('Dthres' in paper)
MaxNeighAllowed=6; % Maximum number of neighbor allowed
Max_myosin_nodes_per_pixels =20;
K=0.5; %Coefficient of myosin pulling
KSp=0.05; %  Actin filamnet spring constant connecting myosin nodes

% Defining folder and printing initial image
ver=0;
folder=strcat('UpdatedCaseTest2020_Tempass');
foldername=folder;
if exist(foldername,'dir')~=7
    mkdir(foldername);
else
    while  exist(foldername, 'dir')==7
        ver= ver+1;
        foldername= strcat(folder,num2str(ver));
    end
    mkdir(foldername)
end
absoluteFolderPath = foldername;
```

----- Myosin Initialization-------------

```
disp('initializing Nodes')
```

```
% %% making node IDs and myosin nodes
Mc_NodeCount=1;
% %%% for circular
cellCenTemp = cellCenters(1,:);
cellCenter_x = cellCenTemp(1,2); %% col
cellCenter_y = - cellCenTemp(1,1); %% row

% % fill passive nodes in the allocated area
for x =cellCenter_x-MyoR : cellCenter_x+ MyoR
    y1= cellCenter_y - sqrt((MyoR)^2- (x-cellCenter_x)^2);
    y2= cellCenter_y + sqrt((MyoR)^2- (x-cellCenter_x)^2);
    for y = y1:y2
        row =round(-y);
        col = round(x);
        rno=rand(1);
        if rno< Den_m              % Density of Myosin Nodes
            Combo(row,col)= Dm;     %  Passive Myosin in combo
        end
    end
end

% %% for circular or SQUARE - clear the area for activating nodes

for x =cellCenter_x-ActM : cellCenter_x+ ActM
%     y1= cellCenter_y - sqrt((ActM)^2- (x-cellCenter_x)^2);
%     y2= cellCenter_y + sqrt((ActM)^2- (x-cellCenter_x)^2);
%     for y = y1:y2
for y= cellCenter_y-ActM-35 : cellCenter_y+ ActM+35
        row =round(-y);
        col = round(x);

            Combo(row,col)= 0;     %  Myosin in combo
     end
end
%


NoOfpassiveMyosin = sum(sum(Combo))/Dm;

NoOfActiveMyosin=0;

% %% ----Fill the allocated area with active nodes
for x =cellCenter_x-ActM : cellCenter_x+ActM
%     y1= cellCenter_y - sqrt(ActM^2- (x-cellCenter_x)^2);
%     y2= cellCenter_y + sqrt((ActM)^2- (x-cellCenter_x)^2);
%     for y = y1:y2
for y= cellCenter_y-ActM-35 : cellCenter_y+ ActM+35
        row =round(-y);
        col = round(x);
        rno=rand(1);
        if rno< Den_m             % Density of Myosin Nodes
            Combo(row,col)= Mc;     %  Active Myosin in combo
            NoOfActiveMyosin=NoOfActiveMyosin+1;
        end
```

```
    end
end


fig = figure;
imagesc(Combo)
NoofMyonodes=0;

NoOfMyosinNodesa = NoofMyonodes;
```

initializing Nodes

% ------making Node IDs--------

```
% Mc_Node is an array that holds the information and flags for each
 node of
% myosin, like its active or passive, its position etc.

Mc_NodeCount=1;
NodeCountPerPixel=zeros(bound,bound);
disp('Making NodeIds')
for colNo=1:bound
    for rowNo=1:bound
        if Combo(rowNo,colNo)>0

            NodeCountPerPixel(rowNo, colNo) = NodeCountPerPixel(rowNo,
 colNo) + 1 ;
            Mc_NodeNo(rowNo,colNo)=Mc_NodeCount;

            Mc_Node(Mc_NodeCount,ROW)=rowNo;
            Mc_Node(Mc_NodeCount,COL)=colNo;
            Mc_Node(Mc_NodeCount,NO_OF_NEIGHBOR)=0; %NoOfNeighbour
            Mc_Node(Mc_NodeCount,COMBOVALUE)=Combo(rowNo,colNo);  %%
 Helpful when testing active passive nodes
            Mc_Node(Mc_NodeCount,MEAN_LENOFCONNEC)=0;  %% helpful for
 calculation of tn_rest dynamically for each simulation
            if Combo(rowNo, colNo)==Mc
            Mc_Node(Mc_NodeCount,MYO_CONC)=Myo_conc;%rand(1);
            else
            Mc_Node(Mc_NodeCount,MYO_CONC)=0;
            end

           if rowNo> (bound-15) | rowNo<15 | colNo>(bound-15) |
 colNo<15
             Mc_Node(Mc_NodeCount,MOVEORNOT)=1;%Moveable NOde
           else
             Mc_Node(Mc_NodeCount,MOVEORNOT)=0;
           end
%

            Mc_NodeCount=Mc_NodeCount+1;
        end
    end
```

```
end
Mc_NodeCount=Mc_NodeCount-1;

%%%%%%---------Initialization Ends---------%%%%%

disp('Making Node Neighbors')
```

-----Making Node neighbors-----------

```
neighborhood = zeros(Mc_NodeCount,MaxNeighAllowed);
%
AvailNeighRegistry=zeros(Mc_NodeCount,1);
RandNodes=randperm(Mc_NodeCount);

for i = 1:Mc_NodeCount
    NodeID= RandNodes(i) ; %randomly slect a myosin node
    AN=0;

    if(Mc_Node(NodeID,4)<MaxNeighAllowed)%check if the #neighbors is
 less than allowed

        rowNo = Mc_Node(NodeID,ROW);
        colNo = Mc_Node(NodeID,COL);


        % % search a circular area for neighbors
        for x= round(rowNo)-
Dis_Thres_Neigh1:round(rowNo)+Dis_Thres_Neigh1
            y1= colNo - sqrt(Dis_Thres_Neigh1^2- (x-round(rowNo))^2);
            y2= colNo + sqrt(Dis_Thres_Neigh1^2- (x-round(rowNo))^2);
            %                   [NodeID x y1 y2]
            for y= y1:y2
                if Mc_NodeNo(round(x),round(y))>0 &&
 Mc_NodeNo(round(x),round(y))~= NodeID %% if you found a neighbor
                    NeighborID = Mc_NodeNo(round(x),round(y));

                    % % check if the neighbor you found is already
                    % listed as neighbor to avoid redundancy

                    alreadyANeighbor = 0;
                    for k= 1:Mc_Node(NodeID,NO_OF_NEIGHBOR)
                        if NeighborID == neighborhood(NodeID,k)
                            alreadyANeighbor=1;
                            break;
                        end
                    end

                    % % add the neighbor in the available neighor
                    % list to be used for selecting neighbors
                    if ((alreadyANeighbor==0) &&
 Mc_Node(NeighborID,4)<(MaxNeighAllowed))
                        AN=AN+1;
                        AvailableNeighbors(NodeID, AN)=NeighborID;
```

```
                    end

                end
            end
        end


        % % check here if Available Neighbors array is formed

        neighborAllowed = MaxNeighAllowed - Mc_Node(NodeID,4);

        if AN>0

            if AN <= neighborAllowed

                for i= 1:AN

                    NeighborID=AvailableNeighbors(NodeID,i);

                    %make connection
                    neighborhood(NodeID,
Mc_Node(NodeID,NO_OF_NEIGHBOR)+1) = NeighborID;%add neighborId to
neighborhood array of selected node
                    neighborhood(NeighborID,
Mc_Node(NeighborID,NO_OF_NEIGHBOR)+1) = NodeID; % add selected nodeId
to neighborhood array of neighbor

                    %update neighborcount of both neighbor and node
                    Mc_Node(NeighborID,NO_OF_NEIGHBOR) =
Mc_Node(NeighborID,NO_OF_NEIGHBOR)+1;
                    Mc_Node(NodeID,NO_OF_NEIGHBOR) =
Mc_Node(NodeID,NO_OF_NEIGHBOR)+1;

                end
            % this is the case when there are more available neighbors
than required and so you have to select which neighbors you will make
connection with
            else
                R = randperm(AN,neighborAllowed);

                % repeat making connections
                for i= 1:neighborAllowed

                    NeighborID=AvailableNeighbors(NodeID,R(i));

                    %make connection
                    neighborhood(NodeID,
Mc_Node(NodeID,NO_OF_NEIGHBOR)+1) = NeighborID;
                    neighborhood(NeighborID,
Mc_Node(NeighborID,NO_OF_NEIGHBOR)+1) = NodeID;
                    %update neighborcount
                    Mc_Node(NeighborID,NO_OF_NEIGHBOR) =
Mc_Node(NeighborID,NO_OF_NEIGHBOR)+1;
```

```
                    Mc_Node(NodeID,NO_OF_NEIGHBOR) =
 Mc_Node(NodeID,NO_OF_NEIGHBOR)+1;

                end
            end
        end
    end


end
```

Following lines calculate the mean resting length of connections

```
 for NodeID = 1: Mc_NodeCount
     Connection_length=0;
     tn_Nb=0;
            for i = 1: Mc_Node(NodeID,NO_OF_NEIGHBOR)  % for all
 neighbors

                NbID = neighborhood(NodeID,i);  % get Neighbor iD

                tn_c_Nb= Mc_Node(NbID,COL)- Mc_Node(NodeID,COL);  %
 OUTWARD VECTOR since pulling from neighbor will be directed out

                tn_r_Nb= Mc_Node(NbID,ROW)- Mc_Node(NodeID,ROW);

                tn_Nb = 0.001+sqrt((tn_r_Nb)^2 + (tn_c_Nb)^2);

                Connection_length= Connection_length+tn_Nb;
            end

            if Mc_Node(NodeID,NO_OF_NEIGHBOR)>0
            Mc_Node(NodeID,MEAN_LENOFCONNEC)=Connection_length/
Mc_Node(NodeID,NO_OF_NEIGHBOR) ;
            else
                Mc_Node(NodeID,MEAN_LENOFCONNEC)=0;
            end
 end
 tn_rest= mean(Mc_Node(:,MEAN_LENOFCONNEC));
```

print an image of initial setup

```
cd (absoluteFolderPath)
filename=strcat('initialImage');
fig = figure;
imagesc(Combo)
% imshow(Cap)
print(fig,filename,'-dpng');
% filename=strcat('initialMyo');
% fig = figure;
```

```
%
% imshow(Myo)
% print(fig,filename,'-dpng');
cd ..
```

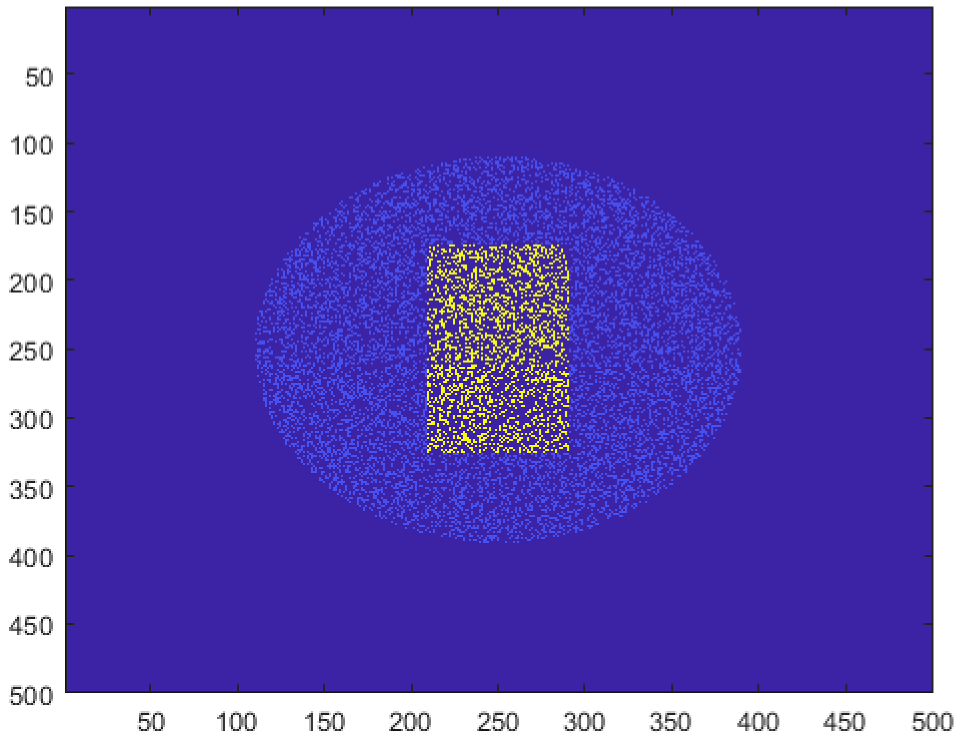

Published with MATLAB® R2020b

Table of Contents

SIMULATION STARTS HERE 1
----------Myosin forces calulation------ 1
If there is tension in the spring 2
----Following lines of code apply the forces calculated above to move the nodes 3
------Visualize the output ------ 4

SIMULATION STARTS HERE

This file is used to run the simulation after the initialization is completed. It first calculates force on individual nodes of actomyosin material and then moves moves nodes based on the force applied, thus implementing Equation 1-4. Finally it generates output in form of images to visualize and study material behavior in the given setup.

```
NodeDensity = zeros(4000,2);
for mcs=1:3500

    mcs


    NewActin=0;

    F_am=zeros(Mc_NodeCount,2);  % net Actomyosin force
    F_r_Nb= zeros(Mc_NodeCount,MaxNeighAllowed); %  Actomyosin force
 in x direction by ith neighbor
    F_c_Nb= zeros(Mc_NodeCount,MaxNeighAllowed);  %  actomyosin force
 in y direction by ith neighbor
    F_r_NbSp= zeros(Mc_NodeCount,MaxNeighAllowed); % Spring force in x
 direction by ith neighbor
    F_c_NbSp= zeros(Mc_NodeCount,MaxNeighAllowed);  % Spring force in
 y direction by ith neighbor
    F_Spring=zeros(Mc_NodeCount,2);     % Net PRing force by all
 nbeighbors
    F_myoT=zeros(Mc_NodeCount,2);
```

----------Myosin forces calulation------

```
    RandNodes=randperm(Mc_NodeCount);
    for i = 1: Mc_NodeCount
        NodeID= RandNodes(i);
        %   %   Calculating forces
        if Mc_Node(NodeID,MOVEORNOT)==1
            %             disp('found a fixed node, so net force is
 zero')
            F_MyoT(NodeID,ROW)= 0;
            F_MyoT(NodeID,COL)= 0;
            % disp('NodeID=')NodeID ;
        else
            % Force calculation of myosin  starts here....
```

```
            for i = 1: Mc_Node(NodeID,NO_OF_NEIGHBOR)  % for all
neighbors

               NbID = neighborhood(NodeID,i);  % get Neighbor iD

               tn_c_Nb= Mc_Node(NbID,COL)- Mc_Node(NodeID,COL);  %
OUTWARD VECTOR since pulling from neighbor will be directed out

               tn_r_Nb= Mc_Node(NbID,ROW)- Mc_Node(NodeID,ROW);

               tn_Nb = 0.001+sqrt((tn_r_Nb)^2 + (tn_c_Nb)^2);   %
distance between node and neighbor (adding .001 to avaoid numerical
instaibility.)
```

If there is tension in the spring

```
        ----Implementing Equation-2 ---------

               F_NbSp=0;
               if tn_Nb>tn_rest

                   % % Spring force calculation

                   F_NbSp = KSp*(tn_Nb- tn_rest); % force generated
due to spring = contstant times spring tension

                   F_r_NbSp(NodeID,i)= F_NbSp*(tn_r_Nb/tn_Nb); %
outward force felt by the node due to tension
                   F_c_NbSp(NodeID,i)= F_NbSp*(tn_c_Nb/tn_Nb);% This
is equal to magnitude*unit vector

               end


               F_Spring(NodeID,ROW)=  F_Spring(NodeID,ROW) +
F_r_NbSp(NodeID,i);%adding forces by all the neighbors
               F_Spring(NodeID,COL)=  F_Spring(NodeID,COL)+
F_c_NbSp(NodeID,i);

               % % Inter-myosin force calculation
                  %   ----Implementing Equation-1 ---------
           % % calculate pulling forces from  myosin neighbors
               F_Nb=0;
               AM_Neighbor= Mc_Node(NbID,MYO_CONC);  %fetch the
myosin activity  on node and neighbor
               AM_BaseNode= Mc_Node(NodeID,MYO_CONC);

               F_Nb =(K*AM_Neighbor*AM_BaseNode); % force magnitude

               F_r_Nb(NodeID,i)=F_Nb*(tn_r_Nb/tn_Nb);  % Actomyosin
force on the node due to ith nieghbor
               F_c_Nb(NodeID,i)=F_Nb*(tn_c_Nb/tn_Nb); % magnitude *
unit vector
```

```
                    F_am(NodeID,ROW)= F_am(NodeID,ROW)+
F_r_Nb(NodeID,i); %%adding forces by all the neighbors
                    F_am(NodeID,COL)= F_am(NodeID,COL)+
F_c_Nb(NodeID,i); %

                end
                F_myoT(NodeID,ROW)= F_am(NodeID,ROW)+F_Spring(NodeID,ROW);
                F_myoT(NodeID,COL)= F_am(NodeID,COL)+F_Spring(NodeID,COL);

            end

        end
```

----Following lines of code apply the forces calculated above to move the nodes

```
        %----- Implementing equation-4------

        Combo = zeros(bound,bound); %% Resetting the lattice


        RandNodes=randperm(Mc_NodeCount);
        for i = 1: Mc_NodeCount
            NodeID= RandNodes(i);

            colNo= Mc_Node(NodeID,COL);
            rowNo= Mc_Node(NodeID,ROW);


            % % rounding to 2 didgit to control spatial resolution
            NewcolNo = round(colNo+ F_myoT(NodeID,COL),2);
            NewrowNo = round(rowNo+ F_myoT(NodeID,ROW),2);

            %%Next each node checks if the space is available or already
            %%saturated by maximum no of nodes
            noOfNodesAtNewPosition = NodeCountPerPixel((round(NewrowNo)),
(round(NewcolNo)));

            if noOfNodesAtNewPosition == Max_myosin_nodes_per_pixels
                NewcolNo= colNo;
                NewrowNo= rowNo;
            end


%%----- Update the variables with new position to implement the
 move---

 Mc_NodeNo(round(Mc_Node(NodeID,ROW)),round(Mc_Node(NodeID,COL)))= -1;
   %% reset the NodeNo in order to create it again afresh
```

```
        Mc_Node(NodeID,ROW)= NewrowNo; %
        Mc_Node(NodeID,COL)= NewcolNo;

        Mc_NodeNo(round(NewrowNo),round(NewcolNo))=NodeID;

        Combo(round(NewrowNo),round(NewcolNo))=
Mc_Node(NodeID,COMBOVALUE); % refresh combo

        NodeCountPerPixel((round(NewrowNo)),(round(NewcolNo))) =
NodeCountPerPixel((round(NewrowNo)),(round(NewcolNo))) + 1;
        NodeCountPerPixel((round(rowNo)), (round(colNo))) =
NodeCountPerPixel((round(rowNo)),(round(colNo))) - 1 ;


    end
    %%%%%%%%%%%%%%%%%%------END--------%%%%%%%%%%%
```

------Visualize the output ------

```
    if (mcs)==2
        close all
        cd (absoluteFolderPath)
        Actinfoldername=strcat('Combo');
        mkdir(Actinfoldername);
        cd (Actinfoldername)
        filename=strcat('Actin_',num2str(mcs));
        fig = figure;
        C=uint8(Combo);
        imshow(C)
        axis square
        print(fig,filename,'-dpng');

        cd ..
        cd ..

        %
    end

    if mod(mcs,100)==0
        close all
        cd (absoluteFolderPath)
        Actinfoldername=strcat('Combo');
        mkdir(Actinfoldername);
        cd (Actinfoldername)
        filename=strcat('Actin_',num2str(mcs));
        fig = figure;
        %                  imagesc(Combo)
        %                  colormap('hot')
        C=uint8(Combo);
        imshow(C)
        %                       imshow(C, 'Colormap', jet(255))
        axis square
        print(fig,filename,'-dpng');
```

```
        cd ..
        cd ..

        %
    end

    %

end
```

Published with MATLAB® R2020b
