# Supplementary material for: Emergence of a smooth interface from growth of a dendritic network against a mechanosensitive contractile material
Source: eLife. 2021 Aug 23;10:e66929. doi: 10.7554/eLife.66929 (PMC8410080; doi:10.7554/eLife.66929)
Supplement: Source code 2. — MATLAB code is available at: https://github.com/medha7575/sharma-et-al-ELIFE2021. [file elife-66929-code2.zip › SharmaetalAnnotatedcodeforArp23ActinAloneModel.pdf]

Table of Contents

This file is used for initialization of the actin nodes and wall position. 1
---------Wall Initialization------------- 1
---------- Initialize Arp 2/3 actin nodes and nucleation points-------- 2

This file is used for initialization of the actin nodes and wall position.

```
clc
clear all
close all

% Following module is called to define different parameters that are
 used
% in this simulation
ParameterDefination
```

---------Wall Initialization-------------

```
disp('initializing Nodes')
Wa_NodeCount=1;

cellCenTemp = cellCenters(1,:);
cellCenter_x = cellCenTemp(1,2); %% col
cellCenter_y = - cellCenTemp(1,1); %% row

%%-- Following lines of code will create a circular wall
for x =cellCenter_x-WalR : cellCenter_x+ WalR
    y1= cellCenter_y - sqrt((WalR)^2- (x-cellCenter_x)^2);
    y2= cellCenter_y + sqrt((WalR)^2- (x-cellCenter_x)^2);
    for y = y1:y2
        row =round(-y);
        col = round(x);

        Combo(row,col)= Wa;
    end
end

for x =cellCenter_x-(CapR+40) : cellCenter_x+ (CapR+40)
    y1= cellCenter_y - sqrt((CapR+40)^2- (x-cellCenter_x)^2);
    y2= cellCenter_y + sqrt((CapR+40)^2- (x-cellCenter_x)^2);
    for y = y1:y2
        row =round(-y);
        col = round(x);

            Combo(row,col)= 0;
     end
```

```
end

disp('Making Wall Nodes')
for colNo=1:bound
    for rowNo=1:bound
        if Combo(rowNo,colNo)>0

            Wa_NodeNo(rowNo,colNo)=Wa_NodeCount;

            Wa_Node(Wa_NodeCount,ROW)=rowNo;
            Wa_Node(Wa_NodeCount,COL)=colNo;
            Wa_Node(Wa_NodeCount,COMBOVALUE)=Combo(rowNo,colNo);  %%
 Helpful when testing active passive nodes

            Wa_NodeCount=Wa_NodeCount+1;
        end
    end
end
Wa_NodeCount=Wa_NodeCount-1;


% --------Wall Initialization complete------------
```

initializing Nodes
Making Wall Nodes

---------- Initialize Arp 2/3 actin nodes and nucleation points--------

```
%%--Following module initialized the actin nodes and nucleation points

InitiateActin
%%---------Initialization process ends here-----------


cd (absoluteFolderPath)
filename=strcat('initialImage');
fig = figure;
imagesc(Combo)
print(fig,filename,'-dpng');
cd ..
```

initializing Npfs
Making NFPIds

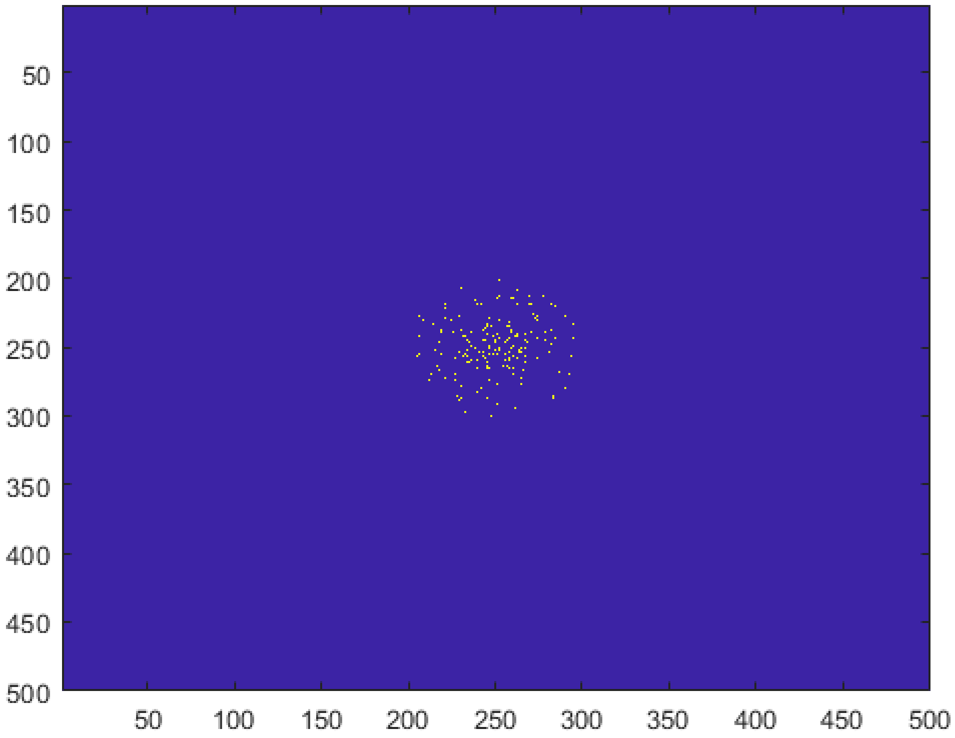
50
100
150
200
250
300
350
400
450
500
50 100 150 200 250 300 350 400 450 500

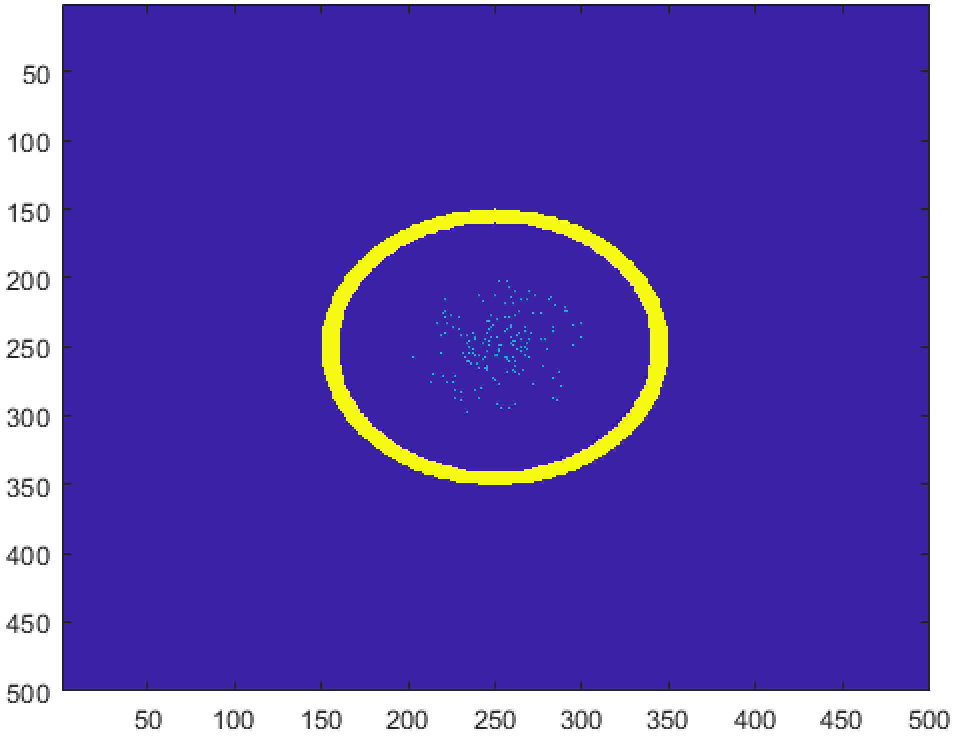
50
100
150
200
250
300
350
400
450
500
50 100 150 200 250 300 350 400 450 500

Published with MATLAB® R2020b

This files stores all the parameter values that are used in the respective simulation.

It also creates the folder in which the data of the simulation will be stored at run time

```
cellCenters=[250,250];

%%-Following are the variables for easily assigning to array while
 initializing

TOTAL_NODES_AT_LOCATION = 3;
NODE_ID_AT_LOCATION=4;

ROW=1;
COL=2;
MYO_CONC=3;
NO_OF_NEIGHBOR=4;
MOVEORNOT=5;
COMBOVALUE=6;
MEAN_LENOFCONNEC=7;

BRANCHORNOT=4;
ASSCNPF=3;
THETA=5;
LEN=6;
MCSATBIRTH=7;
AGE=8;
%%-Following are the parameters used

% Lattice defination
bound=500;
rows=bound;
cols=bound;

Combo = zeros(bound,bound);% Used for visualizing the lattice
npfChart= zeros(bound,bound); % Specifies where the nucleation
 promoters are present
Wal=zeros(bound,bound); % Used for visualizing the Wall activity field



Wa_NodeNo = ones(rows,cols).*-1; % Main array holding Wall
Ac_NodeNo = ones(rows,cols).*-1; % Main array holding actin material
 features

CapR1=20;
```

```
CapR=50;% %initial radius in which npfs are distributed
WalR=100;% % keeping wall farther;

Vnpf=50; %Value assigned to npf in npfChart
Ac= 150 ; % Actin
Wa= 350;


Den_c1=0.1;% initial npf density
Den_c=0.01;

% Other actin material parameters

AgeBr=9;  % age of branching
Knpf=0.5; % 'Kpoly'  or Actin polymerization coefficient
Lth=20; % Max length
AgeTh=20; % age of death
P_del=0.7; %probability of deletion

% Segregation parameters for wall and actin cap

SR=5; %Wall and actin search radius for each other
Aura= 2;% Inter-material Volume Exclusion Threshold ('Vex' )

% Defining folder and printing initial image
ver=0;
folder=strcat('With Wall_BoundaryTest_Den_c_',num2str(Den_c));
foldername=folder;
if exist(foldername,'dir')~=7
    mkdir(foldername);
else
    while  exist(foldername, 'dir')==7
        ver= ver+1;
        foldername= strcat(folder,num2str(ver));
    end
    mkdir(foldername)
end
absoluteFolderPath = foldername;

% % -Following lines of code will set directions used to initialize
 actin from npf. Used to direct
% arp 2/3 actin in different directions

k=1;
for i=-1:1
    for j=-1:1
        if i==0 & j==0
            continue
        else
            directions(k,1)= i; %r
            directions(k,2)= j; %c
            directions(k,3)= rad2deg(cart2pol(j,-i));
            k=k+1;
        end
```

```
    end
end
```

Published with MATLAB® R2020b

This file is called by 'SetUp_Initiate' to initiate Arp2/3 actin nodes and nucleation points

```
disp('initializing Npfs')

%%-----Following lines of code will create a circular random area
%%containing nucleation points

cellCenTemp = cellCenters(1,:);
cellCenter_x= cellCenTemp(1,2); %% col
cellCenter_y= - cellCenTemp(1,1); %% row
for x =cellCenter_x-CapR1 : cellCenter_x+CapR1
    y1= cellCenter_y - sqrt(CapR1^2- (x-cellCenter_x)^2);
    y2= cellCenter_y + sqrt((CapR1)^2- (x-cellCenter_x)^2);
    for y = y1:y2
        row =round(-y);
        col = round(x);
        rno=rand(1);
        if rno< Den_c1             % Density of inner Actin Nodes in
 cap
            npfChart(row,col)= Vnpf;    % Initializing Nucleation
 points
        end
    end
end

for i=1:length(cellCenters)
% %% for circular
cellCenTemp = cellCenters(1,:);
cellCenter_x= cellCenTemp(1,2); %% col
cellCenter_y= - cellCenTemp(1,1); %% row
for x =cellCenter_x-CapR : cellCenter_x+CapR
    y1= cellCenter_y - sqrt(CapR^2- (x-cellCenter_x)^2);
    y2= cellCenter_y + sqrt((CapR)^2- (x-cellCenter_x)^2);
    for y = y1:y2
        row =round(-y);
        col = round(x);
        rno=rand(1);
        if rno< Den_c             % Density of outer Actin Nodes in
 cap
            npfChart(row,col)= Vnpf;    % Initializing Nucleation
 points
        end
    end
end
end

% Count and initialize Nucleation Points
% %
disp('Making NFPIds')
NPFCount=1;
```

```
for colNo=1:bound
    for rowNo=1:bound
        if npfChart(rowNo,colNo)==Vnpf
            NPF(NPFCount,1)=rowNo;%NPF stores nucleation points
 location data
            NPF(NPFCount,2)=colNo;
            NPFCount=NPFCount+1;

        end
    end
end
NPFCount=NPFCount-1;
```

initializing Npfs
Making NFPIds

```
imagesc(npfChart)
pause(1)

%%---Following lines of code will initialize actin nodes using the
%%nucleation points

Ac_NodeCount=1;
for i=1:NPFCount
    row= NPF(i,1);
    col= NPF(i,2);
    dir=randi(8);
    theta = directions(dir,3);
    dir_c=  cos(deg2rad(theta));
    dir_r=  -sin(deg2rad(theta));

    len=randi(8);
    Fnpf_r= Knpf*dir_r*len; %polymerization force
    Fnpf_c= Knpf*dir_c*len;

    rowNo=row+Fnpf_r; % actin position
    colNo=col+Fnpf_c;  % actin position

    Ac_Node(Ac_NodeCount,ROW)=rowNo; %Update Actin array with rowNo
    Ac_Node(Ac_NodeCount,COL)=colNo;
    Ac_Node(Ac_NodeCount,BRANCHORNOT)= 0; % zero at this position
 represents unbranched
    Ac_Node(Ac_NodeCount,THETA)=theta ;%direction of actin filamnet
    Ac_Node(Ac_NodeCount,LEN)= sqrt((rowNo-row)^2+(colNo-col)^2); %
 will store length of filament
    Ac_Node(Ac_NodeCount,ASSCNPF)= i;  % associated NPF
    Ac_Node(Ac_NodeCount,MCSATBIRTH)= 0;  % Simulation step of
 creation like date of birth
    Ac_Node(Ac_NodeCount,AGE)= len;  % age of actin filament is made
 different initially for variability

  Ac_NodeNo(round(rowNo),round(colNo))= Ac_NodeCount;  % this stores
 Actin node ID in an array
```

```
  Combo(round(rowNo),round(colNo))= Ac;  % Update Combo(visualization
 lattice) with Actin

    Ac_NodeCount=Ac_NodeCount+1;
end
Ac_NodeCount=Ac_NodeCount-1;
```

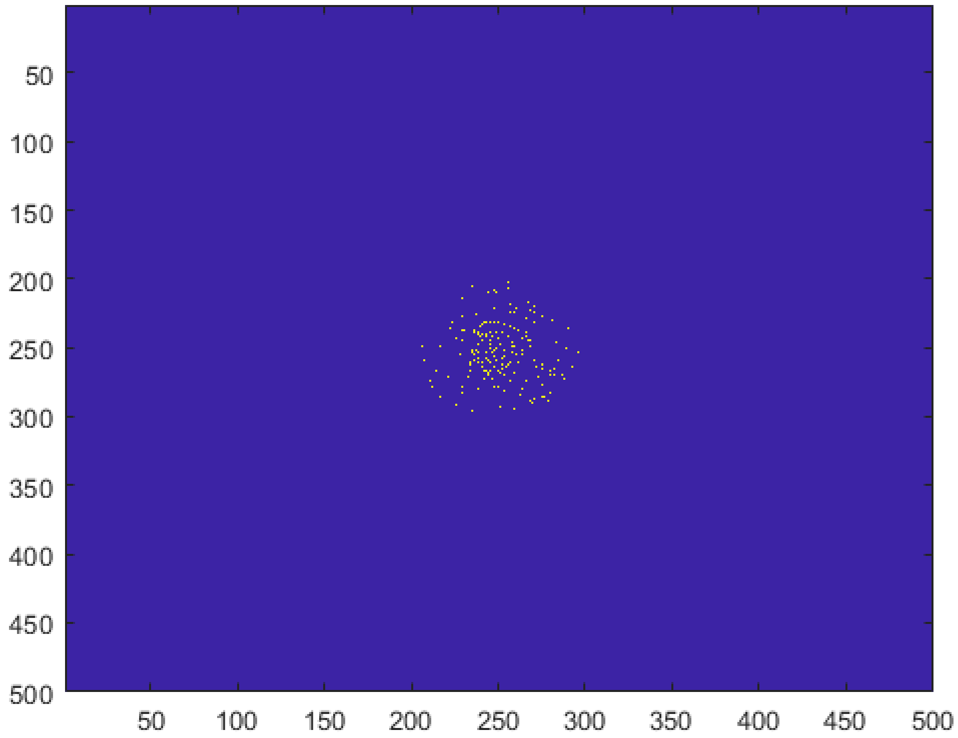

Published with MATLAB® R2020b

Table of Contents

SIMULATION STARTS HERE 1
% ----Forces calulation module----- 1
Following lines of code outputs the images for visualization 2

SIMULATION STARTS HERE

This file is used to run the simulation after the initialization is completed. It calls module 'Force_Calculation' to first calculate force on inidividual nodes of actin material and then moves actin nodes by calling module 'MoveActin'. Thereafter, the code deletes filaments that have aged. It also removes wall to test affect of wall removal. Finally it generates output in form of images to visualize and study material behavior in the given setup.

```
NodeDensity = zeros(4000,2);
for mcs=1:5000

    mcs

    NewActin=0;
    F_AcNet=zeros(Ac_NodeCount,2);

    Combo = zeros(bound,bound);
```

% ----Forces calulation module-----

```
    Force_Calculation;

    %Since lattice was reset, wall is placed again in the following
 lines

    for NodeID = 1: Wa_NodeCount
        colNo= Wa_Node(NodeID,COL);
        rowNo= Wa_Node(NodeID,ROW);
        Combo(round(rowNo),round(colNo))=Wa;
    end
    %---Following Module is called to move actin nodes---
    MoveActin;

    %%%%%%%%%%%%%% For Deleting Filamnets  %%%%%%%%%%%%%%%%%%%%%%%
    kount=0;
    for NodeID= 1:Ac_NodeCount
        if NodeID>Ac_NodeCount
            break
        end
        if Ac_Node(NodeID,AGE) > (AgeTh)
            Rno= rand(1);
            if Rno< P_del
                %             disp('count deleting nodes')
                kount= kount+1;
```

```
                NodeToDel(kount)= NodeID;

            end
        end
    end

    %
    for del = kount:-1:1  %% deleting in reverse order to avoid
shifting overhead of nodeID
        NodeID= NodeToDel(del);

Combo(round(Ac_Node(NodeID,ROW)),round(Ac_Node(NodeID,COL)))=0;
        %     disp('Deleting Now')
        Ac_Node(NodeID,:)=[];
        Ac_NodeCount=Ac_NodeCount-1;

    end

    Ac_NodeNo=ones(rows,cols).*-1;
    for NodeID= 1:Ac_NodeCount

Ac_NodeNo(round(Ac_Node(NodeID,ROW)),round(Ac_Node(NodeID,COL)))=NodeID;
    end

    %%%-------Follwing lines of code will remove the wall at the
assigned
    %%%simulation step

    if mcs==2000%
        % %  remove wall
        for NodeID = 1: Wa_NodeCount

            colNo= Wa_Node(NodeID,2);
            rowNo= Wa_Node(NodeID,1);
            Wa_NodeNo(round(rowNo),round(colNo))=-1;
            Wa_Node(NodeID,3)=0;
        end
        Wa_NodeCount=1;

        Wa_NodeNo(round(1),round(1))=1;
        Wa_Node(1,3)=350;
    end


    %%%%%%%%%%%%%%%%%%-----END--------%%%%%%%%%%%%%%%%%%%%%
```

Following lines of code outputs the images for visualization

```
    if mcs==2
        close all
        cd (absoluteFolderPath)
```

```
        Actinfoldername=strcat('Combo');
        mkdir(Actinfoldername);
        cd (Actinfoldername)
        filename=strcat('Actin_',num2str(mcs));
        fig = figure;
        imagesc(Combo)
        axis square
        colormap('hot')
        print(fig,filename,'-dpng');
        cd ..

        Pointfoldername=strcat('Points');
        mkdir(Pointfoldername);
        cd (Pointfoldername)
        Combo(find(Combo == 350)) = 0;  %%%Keeping wall
        imshow(Combo)
        %

        axis square
        colormap('hot')

        %       imshow(C, 'Colormap', jet(255))
        print(fig,filename,'-dpng');


        cd ..
        cd ..
    end

    if mod(mcs,100)==0
        close all
        cd (absoluteFolderPath)
        Actinfoldername=strcat('Combo');
        mkdir(Actinfoldername);
        cd (Actinfoldername)
        filename=strcat('Combo_',num2str(mcs));
        fig = figure;
        imagesc(Combo)
        axis square
        colormap('hot')
        print(fig,filename,'-dpng');
        cd ..

        Pointfoldername=strcat('Points');
        mkdir(Pointfoldername);
        cd (Pointfoldername)
        Combo(find(Combo == 350)) = 0;
        imshow(Combo)


        axis square
        colormap('hot')

        %       imshow(C, 'Colormap', jet(255))
```

```
        print(fig,filename,'-dpng');

        cd ..
        cd ..

        %
    end
    %
    M=Ac_NodeNo>0;
    nnz(M);
    ActinLenCounter(mcs)=length(find(Ac_Node(:,6)<=AgeBr));
    ActinCounter(mcs)=Ac_NodeCount;
    NewBranches(mcs)=NewActin;
    DelActin(mcs)=kount;

end
cd (absoluteFolderPath)
filename=strcat('ActotalPlot_',num2str(mcs));
fig = figure;
plot(ActinCounter)
print(fig,filename,'-dpng');

filename=strcat('LenPlot_',num2str(mcs));
fig = figure;
plot(ActinLenCounter)
print(fig,filename,'-dpng');

filename=strcat('NewActinPlot_',num2str(mcs));
fig = figure;
plot(NewBranches)
print(fig,filename,'-dpng');

filename=strcat('DelActinPlot_',num2str(mcs));
fig = figure;
plot(DelActin)
print(fig,filename,'-dpng');
cd ..
```

Published with MATLAB® R2020b

This file calculates force on actin nodes due to actin polymerization directed outward from nucleation point to enable actin growth

```
%----Implementing Equation-5------
   RandNodes=randperm(Ac_NodeCount);
for i= 1:Ac_NodeCount

    NodeID= RandNodes(i); % randomly pick up an actin node

    npfID=  Ac_Node(NodeID,ASSCNPF);  %   corresponding nucleation
point

    theta = Ac_Node(NodeID,THETA);% angle of the filament used to
get its direction

    dir_C= cos(deg2rad(theta)); %% x is the col
    dir_R= -sin(deg2rad(theta)); %% taking -ve sign because the y
axis of the image is pointed downwards


    if Ac_Node(NodeID,LEN) > Lth % cap the actin filament after a
length threshold
        Fpoly(NodeID,ROW) =0 ;    % If reached certain length
growth stops.
        Fpoly(NodeID,COL) =0 ;
    else


        Fpoly(NodeID,ROW)= Knpf*dir_R;  % polymerization force
        Fpoly(NodeID,COL)= Knpf*dir_C;
    end
```

net force on actin node

```
    F_AcNet(NodeID,ROW)= Fpoly(NodeID,ROW);
    F_AcNet(NodeID,COL)= Fpoly(NodeID,COL);

end
```

Published with MATLAB® R2020b

This file moves actin node to a new position depending of the force applied on the node.

Table of Contents

-----Implementing equation-6 ------- 1
%%%%%%%% Creating Actin Branches %%%%%%%%%%%%%%%% 2

---When actin filament length reaches a threshold, it stopes further growth of the respective actin node. When actin filament reaches a predefined length, it enables branching of that filament, by creating new actin nodes and nucleation points.

-----Implementing equation-6 -------

```
 RandNodes=randperm(Ac_NodeCount);
   for i = 1: Ac_NodeCount

         NodeID= RandNodes(i);

         npfID=  Ac_Node(NodeID,ASSCNPF);

         % % rounding to 2 digit to control spatial resolution

         NewrowNo=  round(Ac_Node(NodeID,ROW)+
F_AcNet(NodeID,ROW),2); % New actin barbed end position
         NewcolNo=  round(Ac_Node(NodeID,COL)+  F_AcNet(NodeID,COL),2);
 % New actin barbed end position


         for R= NewrowNo-Aura : NewrowNo+Aura   % search all around the
node for myosin
             for C= NewcolNo-Aura:NewcolNo+Aura

                 % % % if there is a wall node in the vicinity of the
new
                 % position, actin node is kept in the old position

                 if Wa_NodeNo(round(R),round(C))>-1  %% check if node
is a wall node
                   NewrowNo=Ac_Node(NodeID,ROW);
                   NewcolNo=Ac_Node(NodeID,COL);
                 end

             end
         end
```

```
        %%%%%%%%%%%%%%%%%-------------------- %%%%%%%%%%
        %%---Updating new actin node features----

Ac_NodeNo(round(Ac_Node(NodeID,ROW)),round(Ac_Node(NodeID,COL)))= -1;
        %          Ac_Node=UpdateNode(NodeID, Ac_Node, NewrowNo,
NewcolNo,Ac,NPF);
        Ac_Node(NodeID,ROW)=NewrowNo;
        Ac_Node(NodeID,COL)=NewcolNo;
        Ac_Node(NodeID,LEN)= sqrt((NewrowNo-
NPF(npfID,1))^2+(NewcolNo- NPF(npfID,2))^2); %length of filament
         Ac_Node(NodeID,AGE)= mcs- Ac_Node(NodeID,MCSATBIRTH);  %% age
of actin updated


Ac_NodeNo(round(Ac_Node(NodeID,ROW)),round(Ac_Node(NodeID,COL)))=NodeID;

        Combo(round(NewrowNo),round(NewcolNo))= Ac;  % Add the barbed
end to the combo lattice
```

%%%%%%%%% Creating Actin Branches %%% %%%%%%%%%%%%%%%%%

```
        if Ac_Node(NodeID,AGE)> AgeBr
            if Ac_Node(NodeID,BRANCHORNOT) ==0 % check if no branch
has been generated out of this branch till now
              %
                theta_Node= Ac_Node(NodeID,THETA); % fetching angle of
the current node
                Rflip= rand(1);
                if Rflip>0.5
                    flip = 1;
                else
                    flip = -1;
                end

                theta_NPF= theta_Node+ flip* 70 ;% degree allignment
of the new branch FIXED 70

                BrPoint_row = (NPF(npfID,ROW)+Ac_Node(NodeID,ROW))/2;
                BrPoint_col = (NPF(npfID,COL)+Ac_Node(NodeID,COL))/2;
%
                dir_C= cos(deg2rad(theta_NPF)); % x is col
                dir_R= -sin(deg2rad(theta_NPF)); % y is negavtive for
image

                Fnpf_c= Knpf*dir_C;
                Fnpf_r= Knpf*dir_R;

                % % rounding to 2 didgit to control spatial
resolution:
```

```
                BRrowNo=round((BrPoint_row+Fnpf_r),2); % branched
actin position
                BRcolNo=round((BrPoint_col+Fnpf_c),2);  % branched
actin position


                 if Wa_NodeNo(round(BRrowNo),round(BRcolNo))> -1  %%
just check for presence of wall on that point
                                    disp('cant branch');
                 else

                Ac_Node(NodeID,BRANCHORNOT) = 1;   %%%now branched
              %----creating new nucleation point for the branch----
                NPFCount=NPFCount+1;   %% new npf

                NPF(NPFCount,ROW)= BrPoint_row;
                NPF(NPFCount,COL)= BrPoint_col;

                Ac_NodeCount=Ac_NodeCount+1;

                % Updating Node array for the newborn actin which just
                % branched
                Ac_Node(Ac_NodeCount,ROW)=BRrowNo;
                Ac_Node(Ac_NodeCount,COL)=BRcolNo;
                Ac_Node(Ac_NodeCount,BRANCHORNOT)=0; %unbranched
                Ac_Node(Ac_NodeCount,THETA)=theta_NPF ;%direction of
new actin filamnet
                Ac_Node(Ac_NodeCount,LEN)= sqrt((BRrowNo-
NPF(NPFCount,1))^2+(BRcolNo-NPF(NPFCount,2))^2);
                Ac_Node(Ac_NodeCount,ASSCNPF)= NPFCount;  % associated
NPF
                Ac_Node(Ac_NodeCount,MCSATBIRTH)= mcs;  % time of
creation
                Ac_Node(Ac_NodeCount,AGE)= mcs-
Ac_Node(Ac_NodeCount,MCSATBIRTH);  % age of actin


Combo(round(Ac_Node(Ac_NodeCount,ROW)),round(Ac_Node(Ac_NodeCount,COL)))=
Ac;

Ac_NodeNo(round(Ac_Node(Ac_NodeCount,ROW)),round(Ac_Node(Ac_NodeCount,COL)))=
Ac_NodeCount;

                NewActin=NewActin+1;
                 end
            end

        end

    end
```

Published with MATLAB® R2020b
