# Supplementary material for: Emergence of a smooth interface from growth of a dendritic network against a mechanosensitive contractile material
Source: eLife. 2021 Aug 23;10:e66929. doi: 10.7554/eLife.66929 (PMC8410080; doi:10.7554/eLife.66929)
Supplement: Source code 3. — MATLAB code is available at: https://github.com/medha7575/sharma-et-al-ELIFE2021. [file elife-66929-code3.zip › SharmaetalAnnotatedcodeforCombinedModel.pdf]

This file is used for initialization of the two materials i.e., defining initial position of actomyosin and actin nodes.

Table of Contents

After defining the node properties, it connects the nodes with neighors to create a connected network. 1
This is the first file to be run followed by 'Simulate_CombinedModel' 1
--------Actomyosin material Initialization------------ 1
---- Nucleation points and actin Initialization by calling 'InitiateActin' module 2
Clear myosin from actin area 2
---Defining Myosin nodes properties ----- 3
------Making Node neighbors------- 4
Following lines calculate the mean resting length of connections 6
print an image of initial setup 7

After defining the node properties, it connects the nodes with neighors to create a connected network.

This is the first file to be run followed by 'Simulate_CombinedModel'

```
clc
% clear all
close all

% Following module is called to define different parameters that are
 used
% in this simulation

ParameterDefination
```

--------Actomyosin material Initialization-------------

```
disp('initializing Nodes')
```

```
cellCenTemp = cellCenters(1,:);
cellCenter_x = 500;% cellCenTemp(1,2); %% col
cellCenter_y =-500;% - cellCenTemp(1,1); %% row


% fill whole area with passive myosin
for x =cellCenter_x-(bound/2-6) : cellCenter_x+ (bound/2-6)  %% 6 px
 are deducted to prevent lattice boundary colision
    for y = cellCenter_y-(bound/2-6) : cellCenter_y+ (bound/2-6)
        row =round(-y);
        col = round(x);
        rno=rand(1);
        if rno< Den_m             % Density of Myosin Nodes
            Combo(row,col)= Dm;    %  Myosin in combo
        end
    end
end

% In the desired area convert passive myosin to active
for x =cellCenter_x-MyoR : cellCenter_x+ MyoR
    y1= cellCenter_y - sqrt((MyoR)^2- (x-cellCenter_x)^2);
    y2= cellCenter_y + sqrt((MyoR)^2- (x-cellCenter_x)^2);
    for y = y1:y2
        row =round(-y);
        col = round(x);
        % Density of Myosin Nodes
        if Combo(row,col)== Dm
            Combo(row,col)= Mc;
        end %  Myosin in combo
    end
end
```

---- Nucleation points and actin Initialization by calling 'InitiateActin' module

```
InitiateActin
```

Clear myosin from actin area

```
for i=1:length(cellCenters)
    cellCenTemp = cellCenters(i,:);
    cellCenter_x = cellCenTemp(1,2); %% col
    cellCenter_y = - cellCenTemp(1,1); %% row

    for x =cellCenter_x-CapR : cellCenter_x+ CapR
        y1= cellCenter_y - sqrt((CapR)^2- (x-cellCenter_x)^2);
        y2= cellCenter_y + sqrt((CapR)^2- (x-cellCenter_x)^2);
        for y = y1:y2
            row =round(-y);
            col = round(x);
```

```
                if  Combo(round(row),round(col))== Ac

                    for r= row -5 : row +5
                        for c= col-5: col+5
                            Combo(r,c)= 0;    %  Myosin in combo
                            Combo(row,col)= Ac;    %  Actin in combo
                        end
                    end
                end
            end
        end
    end
```

---Defining Myosin nodes properties -----

```
    % % making Node IDs

    % Mc_Node is an array that holds the information and flags for each
     node of
    % myosin, like its active or passive, its position etc.

    Mc_NodeCount=1;
    NodeCountPerPixel=zeros(bound,bound);
    disp('Making NodeIds')
    for colNo=1:bound
        for rowNo=1:bound
            if Combo(rowNo,colNo)>0

                NodeCountPerPixel(rowNo, colNo) = NodeCountPerPixel(rowNo,
     colNo) + 1 ; % update the #nodes on the picked pixel
                Mc_NodeNo(rowNo,colNo)=Mc_NodeCount; % Mc_NodeNo will hold
     the node ID of node at that position. right now Count=ID

                Mc_Node(Mc_NodeCount,ROW)=rowNo; %Update Myosin array with
     rowNo
                Mc_Node(Mc_NodeCount,COL)=colNo;  %Update Myosin array
     with colNo
                Mc_Node(Mc_NodeCount,NO_OF_NEIGHBOR)=0; %Update Myosin
     array with NoOfNeighbour
                Mc_Node(Mc_NodeCount,COMBOVALUE)=Combo(rowNo,colNo);  %%
     Helpful when testing active passive nodes
                Mc_Node(Mc_NodeCount,MEAN_LENOFCONNEC)=0;  %% helpful for
     calculation of tn_rest dynamically for each simulation
                if Combo(rowNo, colNo)==Mc
                    Mc_Node(Mc_NodeCount,MYO_CONC)=Myo_conc;%
                else
                    Mc_Node(Mc_NodeCount,MYO_CONC)=0;
                end

                if rowNo> (bound-15) | rowNo<15 | colNo>(bound-15) |
    colNo<15
                    Mc_Node(Mc_NodeCount,MOVEORNOT)=1;%Moveable NOde
```

```
                else
                    Mc_Node(Mc_NodeCount,MOVEORNOT)=0;
                end

                Mc_NodeCount=Mc_NodeCount+1;
            end
        end
    end
    Mc_NodeCount=Mc_NodeCount-1;

    %%%%%%---------Initialization Ends---------%%%%%

    disp('Making Node Neighbors')
```

------Making Node neighbors-------

```
    neighborhood = zeros(Mc_NodeCount,MaxNeighAllowed);
    %
    AvailNeighRegistry=zeros(Mc_NodeCount,1);
    RandNodes=randperm(Mc_NodeCount);

    for i = 1:Mc_NodeCount
        NodeID= RandNodes(i) ; %randomly slect a myosin node
        AN=0;

        if(Mc_Node(NodeID,4)<MaxNeighAllowed) % check if the #neighbors is
     less than allowed

            rowNo = Mc_Node(NodeID,ROW);
            colNo = Mc_Node(NodeID,COL);


            % % search a circular area for neighbors
            for x= round(rowNo)-
    Dis_Thres_Neigh1:round(rowNo)+Dis_Thres_Neigh1
                y1= colNo - sqrt(Dis_Thres_Neigh1^2- (x-round(rowNo))^2);
                y2= colNo + sqrt(Dis_Thres_Neigh1^2- (x-round(rowNo))^2);
                %                   [NodeID x y1 y2]
                for y= y1:y2
                    if Mc_NodeNo(round(x),round(y))>0 &&
     Mc_NodeNo(round(x),round(y))~= NodeID %% if you found a neighbor
                        NeighborID = Mc_NodeNo(round(x),round(y));


                        % % check if the neighbor you found is already
                        % listed as neighbor to avoid redundancy

                        alreadyANeighbor = 0;
                        for k= 1:Mc_Node(NodeID,NO_OF_NEIGHBOR)
                            if NeighborID == neighborhood(NodeID,k)
                                alreadyANeighbor=1;
                                break;
                            end
```

```
                    end

                    % % add the neighbor in the available neighor
                    % list to be used for selecting neighbors
                    if ((alreadyANeighbor==0) &&
Mc_Node(NeighborID,4)<(MaxNeighAllowed))
                        AN=AN+1;
                        AvailableNeighbors(NodeID, AN)=NeighborID;

                    end

                end
            end
        end


        % % check here if Available Neighbors array is formed

        neighborAllowed = MaxNeighAllowed - Mc_Node(NodeID,4); %  get
to know how many neibhors can node form

        if AN>0

            if AN <= neighborAllowed

                for i= 1:AN

                    NeighborID=AvailableNeighbors(NodeID,i);

                    %make connection
                    neighborhood(NodeID,
Mc_Node(NodeID,NO_OF_NEIGHBOR)+1) = NeighborID; %add neighborId to
neighborhood array of selected node
                    neighborhood(NeighborID,
Mc_Node(NeighborID,NO_OF_NEIGHBOR)+1) = NodeID; % add selected nodeId
to neighborhood array of neighbor

                    %update neighborcount of both neighbor and node
                    Mc_Node(NeighborID,NO_OF_NEIGHBOR) =
Mc_Node(NeighborID,NO_OF_NEIGHBOR)+1;
                    Mc_Node(NodeID,NO_OF_NEIGHBOR) =
Mc_Node(NodeID,NO_OF_NEIGHBOR)+1;

                end

            else % this is the case when there are more available
neighbors than required and so you have to select which neighbors you
will make connection with
                R = randperm(AN,neighborAllowed);

                % repeat making connections
                for i= 1:neighborAllowed

                    NeighborID=AvailableNeighbors(NodeID,R(i));
```

```
                    %make connection
                    neighborhood(NodeID,
 Mc_Node(NodeID,NO_OF_NEIGHBOR)+1) = NeighborID;
                    neighborhood(NeighborID,
 Mc_Node(NeighborID,NO_OF_NEIGHBOR)+1) = NodeID;
                    %update neighborcount
                    Mc_Node(NeighborID,NO_OF_NEIGHBOR) =
 Mc_Node(NeighborID,NO_OF_NEIGHBOR)+1;
                    Mc_Node(NodeID,NO_OF_NEIGHBOR) =
 Mc_Node(NodeID,NO_OF_NEIGHBOR)+1;

                end
            end
        end
    end


end
%
```

Following lines calculate the mean resting length of connections

```
count  = 0
csvdata=zeros(Mc_NodeCount*6,7);

for NodeID = 1: Mc_NodeCount
    Connection_length=0;
    tn_Nb=0;
    for i = 1: Mc_Node(NodeID,NO_OF_NEIGHBOR)  % for all neighbors

        NbID = neighborhood(NodeID,i);  % get Neighbor iD

        tn_c_Nb= Mc_Node(NbID,COL)- Mc_Node(NodeID,COL);  % OUTWARD
 VECTOR since pulling from neighbor will be directed out

        tn_r_Nb= Mc_Node(NbID,ROW)- Mc_Node(NodeID,ROW);

        tn_Nb = 0.001+sqrt((tn_r_Nb)^2 + (tn_c_Nb)^2);

        Connection_length= Connection_length+tn_Nb;
        count  = count +1

 csvdata(count,:)=[NodeID,NbID,Mc_Node(NodeID,ROW),Mc_Node(NodeID,COL),Mc_Node(NbI
(tn_Nb-3.5)];

    end

    if Mc_Node(NodeID,NO_OF_NEIGHBOR)>0
        Mc_Node(NodeID,MEAN_LENOFCONNEC)=Connection_length/
Mc_Node(NodeID,NO_OF_NEIGHBOR) ;
```

```
    else
        Mc_Node(NodeID,MEAN_LENOFCONNEC)=0;
    end
end
tn_rest= mean(Mc_Node(:,MEAN_LENOFCONNEC));


cd (absoluteFolderPath)
csvwrite('csvdata0.csv',csvdata(1:count,:));
cd ..

%
% %% deleting actin nodes if simulations are to be done without actin
 cap
% %
% le= length(Ac_Node);
% for NodeID= le:-1:1
%         Ac_Node(NodeID,:)=[];
%         Ac_NodeCount=Ac_NodeCount-1;
% end
% Ac_NodeNo=zeros(bound,bound);
```

print an image of initial setup

```
cd (absoluteFolderPath)
filename=strcat('initialImage');
fig = figure;
imagesc(Combo)
% imshow(Cap)
print(fig,filename,'-dpng');
% filename=strcat('initialMyo');
% fig = figure;
%
% imshow(Myo)
% print(fig,filename,'-dpng');
cd ..
```

Published with MATLAB® R2020b

This files stores all the parameter values that are used in the respective simulation.

Table of Contents

It also creates the folder in which the data of the simulation will be stored at run time 1
Following are the variables for easily assigning to array while initializing 1
Following are the parameters used 1
Other actin material parameters 2
Other actomyosin material parameters 2
Intermaterial parameters 3
Defining folder and printing initial image 3

It also creates the folder in which the data of the simulation will be stored at run time

```
cellCenters= [500,500; 300,500; 700,500; 400,330; 600,330; 400,670;
 600,670; 200,330; 200, 670; 500,840; 800, 670; 800, 330; 500,160];
```

Following are the variables for easily assigning to array while initializing

```
TOTAL_NODES_AT_LOCATION = 3;
NODE_ID_AT_LOCATION=4;

ROW=1;
COL=2;
MYO_CONC=3;
NO_OF_NEIGHBOR=4;
MOVEORNOT=5;
COMBOVALUE=6;
MEAN_LENOFCONNEC=7;

BRANCHORNOT=4;
ASSCNPF=3;
THETA=5;
LEN=6;
MCSATBIRTH=7;
AGE=8;
```

Following are the parameters used

```
% Lattice defination
```

```
bound=1000;
rows=bound;
cols=bound;

Combo = zeros(bound,bound); % Used for visualizing the lattice
npfChart= zeros(bound,bound); % Specifies where the nucleation
 promoters are present
Myo=zeros(bound,bound); % Used for visualizing the myosin activity
 field

Mc_NodeNo = ones(rows,cols).*-1; % Main array holding myosin material
 features
Ac_NodeNo = ones(rows,cols).*-1; % Main array holding actin material
 features


CapR1=20; % Inner radius of actin initialization
CapR=50;% Outer radius of actin initialization
MyoR=400;  % Whole myosin distribution area radius


Vnpf=50; %Value assigned to nucleation point in npfChart, Used for
 initialization
Ac= 150 ; % Actin value used for visualization
Mc= 350; % Active Myosin value for visualization
Dm=200; % Dead Myosin value for visualization


Den_c1=0.1;% initial npf density used for inner radius
Den_c=0.01; % initial npf density used for outer radius
Den_m=0.2; % myosin density
```

Other actin material parameters

```
AgeBr=9;  % age of branching
Knpf=0.5; % 'Kpoly' or Actin polymerization coefficient
Lth=20; % Max length
AgeTh=20; % Age of Depolymerization
P_del=0.7; %probability of deletion
```

Other actomyosin material parameters

```
Myo_conc=0.1; %Myosin activity per node/concnetration ('M'in paper)
Dis_Thres_Neigh1=5;%500nm distance Myosin Connection Search Distance
 ('Dthres' in paper)
MaxNeighAllowed=6; % Maximum number of neighbor allowed
Max_myosin_nodes_per_pixels =20;
K=0.5; %Coefficient of myosin pulling
KSp=0.05; %  Actin filamnet spring constant connecting myosin nodes
```

Intermaterial parameters

```
SR=5; %myosin and actin search radius for each other or Inter-material
 SR
Aura= 2; % Inter-material Volume Exclusion Threshold ('Vex' )
```

Defining folder and printing initial image

```
ver=0;  % used to create a new name for the file. Helpful in
 iterations
folder=strcat('FxHYwithCapDen_c1_',num2str(Den_c1),'Den_c_',num2str(Den_c),'Den_m_
 is where file will save
foldername=folder;
if exist(foldername,'dir')~=7
    mkdir(foldername);
else
    while  exist(foldername, 'dir')==7
        ver= ver+1;
        foldername= strcat(folder,num2str(ver));
    end
    mkdir(foldername)
end
absoluteFolderPath = foldername;

% % Setting directions used to initialize actin from npf. Used to
 direct
% arp 2/3 actin in different directions
k=1;
for i=-1:1
    for j=-1:1
        if i==0 & j==0
            continue
        else
            directions(k,1)= i; %r
            directions(k,2)= j; %c
            directions(k,3)= rad2deg(cart2pol(j,-i));
            k=k+1;
        end
    end
end
```

Published with MATLAB® R2020b

This file is called by 'SetUp_Initiate' to initiate Arp2/3 actin nodes and nucleation points

```
disp('initializing Npfs')

%%-----Following lines of code will create a circular random area
%%containing nucleation points

for i=1:length(cellCenters)
% %% for circular
cellCenTemp = cellCenters(i,:);
cellCenter_x= cellCenTemp(1,2); %% col
cellCenter_y= - cellCenTemp(1,1); %% row
for x =cellCenter_x-CapR1 : cellCenter_x+CapR1
    y1= cellCenter_y - sqrt(CapR1^2- (x-cellCenter_x)^2);
    y2= cellCenter_y + sqrt((CapR1)^2- (x-cellCenter_x)^2);
    for y = y1:y2
        row =round(-y);
        col = round(x);
        rno=rand(1);
        if rno< Den_c1             % Density of inner Actin Nodes in
 cap
            npfChart(row,col)= Vnpf;    % Initializing Nucleation
 points
        end
    end
end
end

for i=1:length(cellCenters)
% %% for circular
cellCenTemp = cellCenters(i,:);
cellCenter_x= cellCenTemp(1,2); %% col
cellCenter_y= - cellCenTemp(1,1); %% row
for x =cellCenter_x-CapR : cellCenter_x+CapR
    y1= cellCenter_y - sqrt(CapR^2- (x-cellCenter_x)^2);
    y2= cellCenter_y + sqrt((CapR)^2- (x-cellCenter_x)^2);
    for y = y1:y2
        row =round(-y);
        col = round(x);
        rno=rand(1);
        if rno< Den_c             % Density of outer Actin Nodes in
 cap
            npfChart(row,col)= Vnpf;    % Initializing Nucleation
 points
        end
    end
end
end

% Count and initialize Nucleation Points
```

```
% %
disp('Making NFPIds')
NPFCount=1;

for colNo=1:bound
    for rowNo=1:bound
        if npfChart(rowNo,colNo)==Vnpf
            NPF(NPFCount,1)=rowNo;%NPF stores nucleation points
 location data
            NPF(NPFCount,2)=colNo;
            NPFCount=NPFCount+1;

        end
    end
end
NPFCount=NPFCount-1;
```

initializing Npfs
Making NFPIds

```
imagesc(npfChart)
pause(1)

%%---Following lines of code will initialize actin nodes using the
%%nucleation points

Ac_NodeCount=1;
for i=1:NPFCount
    row= NPF(i,1);
    col= NPF(i,2);
    dir=randi(8);
    theta = directions(dir,3);
    dir_c=  cos(deg2rad(theta));
    dir_r=  -sin(deg2rad(theta));

    len=randi(8);
    Fnpf_r= Knpf*dir_r*len;%polymerization force
    Fnpf_c= Knpf*dir_c*len;

    rowNo=row+Fnpf_r; % actin position
    colNo=col+Fnpf_c;  % actin position

    Ac_Node(Ac_NodeCount,ROW)=rowNo; %Update Actin array with rowNo
    Ac_Node(Ac_NodeCount,COL)=colNo;
    Ac_Node(Ac_NodeCount,BRANCHORNOT)= 0; % A zero at this position
 represents unbranched
    Ac_Node(Ac_NodeCount,THETA)=theta ;%direction of actin filamnet
    Ac_Node(Ac_NodeCount,LEN)= sqrt((rowNo-row)^2+(colNo-col)^2); %
 will store length of filament
    Ac_Node(Ac_NodeCount,ASSCNPF)= i;  % associated NPF
    Ac_Node(Ac_NodeCount,MCSATBIRTH)= 0;  % Simulation step of
 creation like a date of birth
    Ac_Node(Ac_NodeCount,AGE)= len;  % age of actin filament is made
 different initially for variability
```

```
  Ac_NodeNo(round(rowNo),round(colNo))= Ac_NodeCount;  % this stores
 Actin node ID in an array
   Combo(round(rowNo),round(colNo))= Ac;  % Update Combo(visualization
 lattice) with Actin

    Ac_NodeCount=Ac_NodeCount+1;
end
Ac_NodeCount=Ac_NodeCount-1;
```

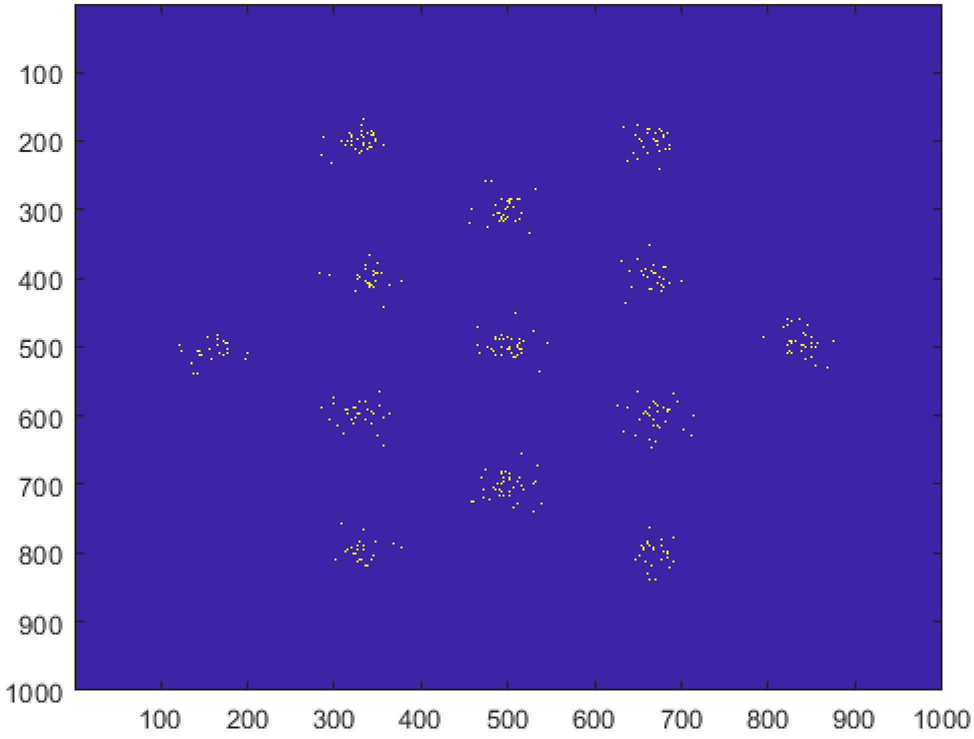

Published with MATLAB® R2020b

Table of Contents

SIMULATION STARTS HERE 1
initializing the arrays that are used in simulation 1
% Forces calculation --- call associated module 1
VISUALIZATION and GENERATING IMAGES%%%%% 3
visualization of myosin concentration 3

SIMULATION STARTS HERE

This file is used to run the simulation after the initialization is completed. It calls module 'Force_Calculation' to first calculate forces on inidividual nodes of both material and then moves actin nodes by calling module 'MoveActin' and myosin nodes by calling module "MoveMysoin". These modules are called randomly, so that both the materials have a random chance to get the first move. Thereafter, the code deletes filaments that have aged. It also removes wall to test affect of wall removal. Finally it generates output in form of images to visualize and study material behavior in the given setup.

```
NodeDensity = zeros(4000,2);
for mcs=1:2500

     mcs
```

initializing the arrays that are used in simulation

```
    NewActin=0;


    F_AcNet=zeros(Ac_NodeCount,2);

    Rho_myo=zeros(Ac_NodeCount,2);
    F_MyoNet=zeros(Mc_NodeCount,2); % total(spring + myosin) force on
 each node
    F_am=zeros(Mc_NodeCount,2);  % net Actomyosin force
    F_r_Nb= zeros(Mc_NodeCount,MaxNeighAllowed); %  Actomyosin force
 in x direction by ith neighbor
    F_c_Nb= zeros(Mc_NodeCount,MaxNeighAllowed);  %  actomyosin force
 in y direction by ith neighbor
    F_r_NbSp= zeros(Mc_NodeCount,MaxNeighAllowed); % Spring force in x
 direction by ith neighbor
    F_c_NbSp= zeros(Mc_NodeCount,MaxNeighAllowed);  % Spring force in
 y direction by ith neighbor
    F_Spring=zeros(Mc_NodeCount,2);     % Net PRing force by all
 nbeighbors
    F_myoT=zeros(Mc_NodeCount,2);
```

% Forces calculation --- call associated module

```
       Force_MyosinThenActinNEW;
```

```
   Combo = zeros(bound,bound);
    R=rand(1);

 %% %Randomly chose actin or mysoin material for moving nodes

   if R>0.5
       MoveMyosin;
       MoveActin;
   else
       MoveActin;
       MoveMyosin;
   end


    %%%%%%%%%%%%%% For Deleting aged Filamnets  %%%%%%%%%%%%%%%%%%%%%
%%
    kount=0;
    for NodeID= 1:Ac_NodeCount
        if NodeID>Ac_NodeCount
            break
        end
        if Ac_Node(NodeID,AGE) > (AgeTh)  %% instead of length compare
 age of actin
            Rno= rand(1);
            if Rno< P_del
                %              disp('count deleting nodes')
                kount= kount+1;
                NodeToDel(kount)= NodeID;

            end
        end
    end

for del = kount:-1:1  %% deleting in reverse order to avoid shifting
 overhead of nodeID
    NodeID= NodeToDel(del);
    Combo(round(Ac_Node(NodeID,ROW)),round(Ac_Node(NodeID,COL)))=0;
    %     disp('Deleteing Now')
    Ac_Node(NodeID,:)=[];
    Ac_NodeCount=Ac_NodeCount-1;

end
% % Reassigning Ac_NodeNo
Ac_NodeNo=ones(rows,cols).*-1;
for NodeID= 1:Ac_NodeCount

 Ac_NodeNo(round(Ac_Node(NodeID,ROW)),round(Ac_Node(NodeID,COL)))=NodeID;
end

    %%%%%%%%%%%%%%%%%%-----THE END--------%%%%%%%%%%%%%%%%%%%%%
```

VISUALIZATION and GENERATING IMAGES%%%%%

```
            if mcs==2

               close all
               cd (absoluteFolderPath)
               Actinfoldername=strcat('Combo');
               mkdir(Actinfoldername);
               cd (Actinfoldername)
               filename=strcat('Actin_',num2str(mcs));
               fig = figure;
               imagesc(Combo)
               axis square
               colormap('hot')

       % % %             imshow(C, 'Colormap', jet(255))
               print(fig,filename,'-dpng');

               cd ..
```

visualization of myosin concentration

```
               MyoCON=zeros(bound,bound);
               for i = 1:Mc_NodeCount
                    roNo= round(Mc_Node(i ,ROW));
                    coNo= round(Mc_Node(i ,COL));
                    valu= Mc_Node(i ,MYO_CONC);
                    MyoCON(roNo,coNo)=valu;
               end
               Myofoldername=strcat('MyoCon');
               mkdir(Myofoldername);
               cd (Myofoldername)

               filename=strcat('MyoCON',num2str(mcs));
               fig = figure;
               imagesc(MyoCON)
            colormap('gray')
               print(fig,filename,'-dpng');



               cd ..
               cd ..

            end

       %     if mod(mcs,100)==0
       %
       %         tbl=tabulate( Mc_Node(:,NO_OF_NEIGHBOR));
       %         cd (absoluteFolderPath)
       %         filename=strcat('FreqN_',num2str(mcs));
```

```
%          fig = figure;
%          bar(tbl(:,1),tbl(:,2))
%           print(fig,filename,'-dpng');
%          cd ..
%      end

    if mod(mcs,50)==0
        close all
        cd (absoluteFolderPath)
        Actinfoldername=strcat('Combo');
        mkdir(Actinfoldername);
        cd (Actinfoldername)
        filename=strcat('Actin_',num2str(mcs));
        fig = figure;
        imagesc(Combo)
        axis square
        colormap('hot')

        %       imshow(C, 'Colormap', jet(255))
        print(fig,filename,'-dpng');

        cd ..


        MyoCON=zeros(bound,bound);
        for i = 1:Mc_NodeCount
             roNo= round(Mc_Node(i ,ROW));
             coNo= round(Mc_Node(i ,COL));
             valu= Mc_Node(i ,MYO_CONC);
             MyoCON(roNo,coNo)=valu;
        end
        cd (Myofoldername)
        filename=strcat('MyoCON',num2str(mcs));
        fig = figure;
        imagesc(MyoCON)
        colormap('gray')
        print(fig,filename,'-dpng');


        cd ..
        cd ..

%
    end
    %
    M=Ac_NodeNo>0;
    nnz(M);
 ActinLenCounter(mcs)=length(find(Ac_Node(:,6)<=AgeBr));
 ActinCounter(mcs)=Ac_NodeCount;
 NewBranches(mcs)=NewActin;
 DelActin(mcs)=kount;

end
   cd (absoluteFolderPath)
```

```
filename=strcat('ActotalPlot_',num2str(mcs));
fig = figure;
plot(ActinCounter)
 print(fig,filename,'-dpng');

filename=strcat('LenPlot_',num2str(mcs));
fig = figure;
plot(ActinLenCounter)
 print(fig,filename,'-dpng');

     filename=strcat('NewActinPlot_',num2str(mcs));
fig = figure;
plot(NewBranches)
 print(fig,filename,'-dpng');

     filename=strcat('DelActinPlot_',num2str(mcs));
fig = figure;
plot(DelActin)
 print(fig,filename,'-dpng');
cd ..
```

Published with MATLAB® R2020b

This file calculates forces first on myosin nodes and then on actin nodes.

Table of Contents

Myosin node experience forces from neighboring nodes and due to actin pushing. 1
Actin node experiences forces from actin polymerization directed outward from nucleation point to enable actin growth and from myosin pushing. 1
---- This section calculates myosin forces ----- 1
If there is tension in the spring 2
--- This section calculates actin forces ----- 5

Myosin node experience forces from neighboring nodes and due to actin pushing.

Actin node experiences forces from actin polymerization directed outward from nucleation point to enable actin growth and from myosin pushing.

---- This section calculates myosin forces -----

```
count  = 0
csvdata=zeros(Mc_NodeCount*6,7);

RandNodes=randperm(Mc_NodeCount);
for i = 1: Mc_NodeCount
    NodeID= RandNodes(i); %randomly pick up one myosin node
    %   %   Calculating forces
    if Mc_Node(NodeID,MOVEORNOT)==1 % check if it is a unmovable node
        %             disp('found a fixed node, so net force is zero')
        F_MyoNet(NodeID,ROW)= 0;  % set all forces on this node to be
 zero
        F_MyoNet(NodeID,COL)= 0;
        % disp('NodeID=')NodeID ;
    else
        % Force calculation of myosin  starts here sicne these are
 movable nodes

        for i = 1: Mc_Node(NodeID,NO_OF_NEIGHBOR)  % for all neighbors
```

```
            NbID = neighborhood(NodeID,i);  % pick one neighbor and
get Neighbor iD
            if NbID ~=0    %Neigbor ID can not be zero,

                tn_c_Nb= Mc_Node(NbID,COL)- Mc_Node(NodeID,COL);  %
OUTWARD VECTOR since pulling from neighbor will be directed out

                tn_r_Nb= Mc_Node(NbID,ROW)- Mc_Node(NodeID,ROW);

                tn_Nb = 0.001+sqrt((tn_r_Nb)^2 + (tn_c_Nb)^2);   %
distance between node and neighbor (adding .001 to avoid numerical
instaibility)

                 if mod(mcs,500)==0
                    count  = count +1

csvdata(count,:)=[NodeID,NbID,Mc_Node(NodeID,ROW),Mc_Node(NodeID,COL),Mc_Node(NbI
(tn_Nb-3.5)];

                end
```

If there is tension in the spring

```
----Implementing Equation-2 ---------

                F_NbSp=0;
                if tn_Nb>tn_rest

                    % % Spring force calculation


                    F_NbSp = KSp*(tn_Nb- tn_rest); % force generated
due to spring = contstant times stretch

                    F_r_NbSp(NodeID,i)= F_NbSp*(tn_r_Nb/tn_Nb); %
outward force felt by the node due to tension
                    F_c_NbSp(NodeID,i)= F_NbSp*(tn_c_Nb/tn_Nb); % This
is equal to magnitude*unit vector

                end


                F_Spring(NodeID,ROW)=  F_Spring(NodeID,ROW) +
F_r_NbSp(NodeID,i); %adding forces by all the neighbors
                F_Spring(NodeID,COL)=  F_Spring(NodeID,COL)+
F_c_NbSp(NodeID,i); % Net Spring force

                % % Inter-myosin force calculation
                %   ----Implementing Equation-1 ---------
                % % calculate pulling forces from  myosin neighbors
                F_Nb=0;
                AM_Neighbor= Mc_Node(NbID,MYO_CONC);% %fetch the
myosin activity  on node and neighbor
                AM_BaseNode= Mc_Node(NodeID,MYO_CONC);%
```

```
                F_Nb =(K*AM_Neighbor*AM_BaseNode); ; % force magnitude

                F_r_Nb(NodeID,i)=F_Nb*(tn_r_Nb/tn_Nb);  % Actomyosin
force on the node due to ith nieghbor
                F_c_Nb(NodeID,i)=F_Nb*(tn_c_Nb/tn_Nb);  % magnitude *
unit vector


                F_am(NodeID,ROW)= F_am(NodeID,ROW)+
F_r_Nb(NodeID,i); % %adding forces by all the neighbors
                F_am(NodeID,COL)= F_am(NodeID,COL)+
F_c_Nb(NodeID,i); %  Net inter-myosin pulling force

            end

        end


        %%-----Following lines of code will calculate actin pushing
forces on myosin due to the actin polymerization----%%%%%%
         %   ----Implementing Equation-7 ---------
        totalPoints=0;
        points=0;

        colNo= Mc_Node(NodeID,COL); % the position of current node
        rowNo= Mc_Node(NodeID,ROW);
        for R= rowNo-SR : rowNo+SR   %%% searching around the Myosin
node in search radius
            for C= colNo-SR:colNo+SR
                if Ac_NodeNo(round(R),round(C))> 0 % if there is an
actin node in vicinity (search radius)
                    totalPoints= totalPoints+1;
                    points(totalPoints,1) = R; %% Save the position
where actin node is present
                    points(totalPoints,2) = C;
                end
            end
        end

        %---Following lines will calculate forces from actin nodes
found in the
        %---above determined positions

        Fcap_R=0;
        Fcap_C=0;

        for i= 1: totalPoints
            R= points(i,1) ;
            C= points(i,2);

            Act2Myo_r= rowNo - R;
            Act2Myo_c= colNo - C;  % vector directed towards the
myosin node
```

```
            tn_Act2Myo= sqrt((Act2Myo_r)^2+(Act2Myo_c)^2);

            % accessing the features of actin - its ID, nucleation
 point and
            % direction
            ActID=  Ac_NodeNo(round(R),round(C));
            npfID=  Ac_Node(ActID,ASSCNPF);
            theta = Ac_Node(ActID,THETA);

            dir_C= cos(deg2rad(theta)); %% x is the col
            dir_R= -sin(deg2rad(theta));

            Fpush_R=Knpf*dir_R;
            Fpush_C=Knpf*dir_C ;  %% forces in x direction
            Fpush = sqrt((Fpush_R)^2+(Fpush_C)^2); % magnitude of
 actin pushing force

            cosPhi= (Act2Myo_r*Fpush_R+Act2Myo_c*Fpush_C)/
(tn_Act2Myo*Fpush);  % calculate cosPhi

            if cosPhi>=0  % if cosPhi>0 --> apply forces else dont
 apply

                Fcap_R(i)=  Fpush_R*cosPhi;
                Fcap_C(i)=  Fpush_C*cosPhi;
            else

                Fcap_R(i)=  0;
                Fcap_C(i)=  0;

            end

        end

        Fcappush(NodeID,ROW)=sum(Fcap_R); % sum up the different actin
 node forces
        Fcappush(NodeID,COL)=sum(Fcap_C);


        %%%%%%----- Net FORCES APPLIED TO MYOSIN NODE -------%%%%%%%%%
        F_myoT(NodeID,ROW)= F_am(NodeID,ROW)+F_Spring(NodeID,ROW);   %
 these are only through myosin material
        F_myoT(NodeID,COL)= F_am(NodeID,COL)+F_Spring(NodeID,COL);

        F_MyoNet(NodeID,ROW)= F_am(NodeID,ROW)+F_Spring(NodeID,ROW)+
 Fcappush(NodeID,ROW); % These are total forces from both materials
        F_MyoNet(NodeID,COL)= F_am(NodeID,COL)+F_Spring(NodeID,COL)+
 Fcappush(NodeID,COL);


    end

end
if mod(mcs,500)==0
```

```
    cd (absoluteFolderPath)
    filename=strcat('csvdata_',num2str(mcs),'.csv');

    csvwrite(filename,csvdata(1:count,:));
    cd ..
end
```

--- This section calculates actin forces -----

```
%----Implementing Equation-5------
%%---Following lines calculate actin polymerization force directed
 outward
%%---from nucelation points.

RandNodes=randperm(Ac_NodeCount);
for i= 1:Ac_NodeCount
    NodeID= RandNodes(i); % randomly pick up an actin node

    npfID=  Ac_Node(NodeID,ASSCNPF);  %  corresponding nucleation
 point

    theta = Ac_Node(NodeID,THETA); % angle of the filament used to get
 its direction

    dir_C= cos(deg2rad(theta)); %% x is the col
    dir_R= -sin(deg2rad(theta)); %% taking -ve sign because the y axis
 of the image is pointed downwards

    if Ac_Node(NodeID,LEN) > Lth % cap the actin filament after a
 length threshold
        Fpoly(NodeID,ROW) =0 ;    % If reached certain length growth
 stops.
        Fpoly(NodeID,COL) =0 ;
    else

        Fpoly(NodeID,ROW)= Knpf*dir_R;  % polymerization force
        Fpoly(NodeID,COL)= Knpf*dir_C;
    end

    %%----Following lines calculate forces from the myosin node
 directed towards actin node----%%%%%%
     %   ----Implementing Supplementary Equation-1 ---------
    totalPoints=0;
    points=0;
    noOfNodesPosition=0;

    colNo= Ac_Node(NodeID,COL); %get the position of actin node
    rowNo= Ac_Node(NodeID,ROW);
    for R= rowNo-SR : rowNo+SR   %%% searching around the actin node
 in search radius
        for C= colNo-SR:colNo+SR
            if Mc_NodeNo(round(R),round(C))> 0  %% if myosin node is
 found in vicinity
```

```
                totalPoints= totalPoints+1;
                points(totalPoints,1) = R; %% Save the position of
myosin
                points(totalPoints,2) = C;
                noOfNodesPosition(totalPoints) =
NodeCountPerPixel((round(R)),(round(C))); % check how many nodes are
actually present at this location
            end
        end
    end

    %---Following lines will calculate forces from myosin nodes found
in the
    %---above determined positions

    RhoMyo_R=0;
    RhoMyo_C=0;
    noOfNodesPosition;
    for i= 1: totalPoints
        R= points(i,1) ;
        C=points(i,2);

        Myo2Act_r= rowNo - R;
        Myo2Act_c= colNo - C;  % vector directed towards the actin
from myo
        tn_Myo2Act= sqrt((Myo2Act_c)^2+(Myo2Act_r)^2);


        MyoID=  Mc_NodeNo(round(R),round(C));

        F_myoR=F_myoT(MyoID,ROW);  % % forces in -y direction
        F_myoC=F_myoT(MyoID,COL);  %% forces in x direction
        F_myo= sqrt((F_myoR)^2+(F_myoC)^2);  %% net magnitude of force
from myosin based on actomyosin material forces

        cosPhi= (Myo2Act_r*F_myoR+Myo2Act_c*F_myoC)/
(tn_Myo2Act*F_myo);

        if cosPhi>=0  % force will be applied only if cosPhi>0

            RhoMyo_R(i)= noOfNodesPosition(totalPoints)*
F_myoR*cosPhi; %force magnitude times the # of nodes at the position
            RhoMyo_C(i)= noOfNodesPosition(totalPoints)*
F_myoC*cosPhi; % gives an assesment of cumulative effect of all nodes
in that position
        else
            RhoMyo_R(i)= 0;
            RhoMyo_C(i)=  0;

        end

    end
    Rho_dynR(NodeID,1)=sum(RhoMyo_R);  % summing up all myo node
forces
```

```
    Rho_dynC(NodeID,1)=sum(RhoMyo_C);


    Rho_myo(NodeID,ROW)= Rho_dynR(NodeID,1);%
    Rho_myo(NodeID,COL)= Rho_dynC(NodeID,1);%


    %%%%%%----- Net FORCES APPLIED TO ACTIN NODE -------%%%%%%%%%
    F_AcNet(NodeID,ROW)= Fpoly(NodeID,ROW)+Rho_myo(NodeID,ROW);
    F_AcNet(NodeID,COL)= Fpoly(NodeID,COL)+Rho_myo(NodeID,COL);

end
```

Published with MATLAB® R2020b

This file moves actin node to a new position depending of the force applied on the node.

Table of Contents

-----Implementing equation-6 ------- 1
%%%%%%%% Creating Actin Branches %%%%%%%%%%%%%%%%% 2

---When actin filament length reaches a threshold, it stopes further growth of the respective actin node. When actin filament reaches a predefined length, it enables branching of that filament, by creating new actin nodes and nucleation points.

-----Implementing equation-6 -------

```
RandNodes=randperm(Ac_NodeCount);
for i = 1: Ac_NodeCount

    NodeID= RandNodes(i);

    npfID=  Ac_Node(NodeID,ASSCNPF);

    % % rounding to 2 digit to control spatial resolution

    NewrowNo=  round(Ac_Node(NodeID,ROW)+  F_AcNet(NodeID,ROW),2); %
 New actin barbed end position
    NewcolNo=  round(Ac_Node(NodeID,COL)+  F_AcNet(NodeID,COL),2);  %
 New actin barbed end position


    for R= NewrowNo-Aura : NewrowNo+Aura   % search all around the
 node for myosin
        for C= NewcolNo-Aura:NewcolNo+Aura

            % % % if there is a myosin node in the vicinity of the new
            % position, actin node is kept in the old position
            if Mc_NodeNo(round(R),round(C))>-1  %% check if node is a
 myosin node
                NewrowNo=Ac_Node(NodeID,ROW);
                NewcolNo=Ac_Node(NodeID,COL);
            end

        end
    end

    %%---Updating actin node features----
    Ac_NodeNo(round(Ac_Node(NodeID,ROW)),round(Ac_Node(NodeID,COL)))=
 -1;
```

```
   Ac_Node(NodeID,ROW)=NewrowNo;
   Ac_Node(NodeID,COL)=NewcolNo;
   Ac_Node(NodeID,LEN)= sqrt((NewrowNo- NPF(npfID,1))^2+(NewcolNo-
NPF(npfID,2))^2); %length of filament
   Ac_Node(NodeID,AGE)= mcs- Ac_Node(NodeID,MCSATBIRTH);  %% age of
actin updated


Ac_NodeNo(round(Ac_Node(NodeID,ROW)),round(Ac_Node(NodeID,COL)))=NodeID;

   Combo(round(NewrowNo),round(NewcolNo))= Ac;  % Add the barbed end
to the combo lattice
```

%%%%%%%%% Creating Actin Branches %%% %%%%%%%%%%%%%%%

```
   if Ac_Node(NodeID,AGE)> AgeBr

       if Ac_Node(NodeID,BRANCHORNOT) ==0 %check if no branch has
been generated out of this branch till now

           theta_Node= Ac_Node(NodeID,THETA); % fetching angle of the
current node
           Rflip= rand(1);
           if Rflip>0.5
               flip = 1;
           else
               flip = -1;
           end

           theta_NPF= theta_Node+ flip* 70 ;% degree allignment of
the new branch FIXED 70

           BrPoint_row = (NPF(npfID,ROW)+Ac_Node(NodeID,ROW))/2;
           BrPoint_col = (NPF(npfID,COL)+Ac_Node(NodeID,COL))/2;
           %
           dir_C= cos(deg2rad(theta_NPF)); % x is col
           dir_R= -sin(deg2rad(theta_NPF)); % y is negavtive for
image

           Fnpf_c= Knpf*dir_C;
           Fnpf_r= Knpf*dir_R;

           % % rounding to 2 didgit to control spatial resolution:

           BRrowNo=round((BrPoint_row+Fnpf_r),2); % branched actin
position
           BRcolNo=round((BrPoint_col+Fnpf_c),2);  % branched actin
position
```

```
            if Mc_NodeNo(round(BRrowNo),round(BRcolNo))> -1  %% just
 check for presence of mysoin on that point
                disp('cant branch');
            else

                Ac_Node(NodeID,BRANCHORNOT) = 1;   %%%now branched
                %----creating new nucleation point for the branch----
                NPFCount=NPFCount+1;   %% new npf

                NPF(NPFCount,ROW)= BrPoint_row;
                NPF(NPFCount,COL)= BrPoint_col;

                Ac_NodeCount=Ac_NodeCount+1;

                % Updating Node array for the newborn actin which just
                % branched
                Ac_Node(Ac_NodeCount,ROW)=BRrowNo;
                Ac_Node(Ac_NodeCount,COL)=BRcolNo;
                Ac_Node(Ac_NodeCount,BRANCHORNOT)=0; %unbranched
                Ac_Node(Ac_NodeCount,THETA)=theta_NPF ;%direction of
 new actin filamnet
                Ac_Node(Ac_NodeCount,LEN)= sqrt((BRrowNo-
NPF(NPFCount,1))^2+(BRcolNo-NPF(NPFCount,2))^2);
                Ac_Node(Ac_NodeCount,ASSCNPF)= NPFCount;  % associated
 NPF
                Ac_Node(Ac_NodeCount,MCSATBIRTH)= mcs;  % time of
 creation
                Ac_Node(Ac_NodeCount,AGE)= mcs-
Ac_Node(Ac_NodeCount,MCSATBIRTH);  % age of actin


 Combo(round(Ac_Node(Ac_NodeCount,ROW)),round(Ac_Node(Ac_NodeCount,COL)))=
 Ac;

 Ac_NodeNo(round(Ac_Node(Ac_NodeCount,ROW)),round(Ac_Node(Ac_NodeCount,COL)))=
 Ac_NodeCount;

                NewActin=NewActin+1;

            end
        end

    end

end
```

Published with MATLAB® R2020b

Table of Contents

This file moves actin node to a new position depending of the force applied on the node. 1
Lastly, this file applies the elevated myosin upon saturation. Thus testing the hypothesis. 1
-----Implementing Equation-4----- 1
if trying to reach a staurated point then Increase myosin 2

This file moves actin node to a new position depending of the force applied on the node.

```
     %----When moving a mysoin node, it checks for availability of
the
     %----new position in two checks. 1. Node/pixel saturation 2.
actin node
     %----occupancy.
```

Lastly, this file applies the elevated myosin upon saturation. Thus testing the hypothesis.

-----Implementing Equation-4-----

```
        RandNodes=randperm(Mc_NodeCount);
    for i = 1: Mc_NodeCount

        NodeID= RandNodes(i);

        colNo= Mc_Node(NodeID,COL);
        rowNo= Mc_Node(NodeID,ROW);

        % % rounding to 2 didgit to control spatial resolution
        NewcolNo = round(colNo+ F_MyoNet(NodeID,COL),2);
        NewrowNo = round(rowNo+ F_MyoNet(NodeID,ROW),2);

        %%---check-1:  Node/pixel saturation

         noOfNodesAtNewPosition = NodeCountPerPixel((round(NewrowNo)),
(round(NewcolNo)));

        if noOfNodesAtNewPosition == Max_myosin_nodes_per_pixels
                    NewcolNo= colNo;
                    NewrowNo= rowNo;
        end
        %%--- Check-2: Actin node occupancy: checking if there is
 actin in the way

        for R= NewrowNo-Aura:NewrowNo+Aura
            for C= NewcolNo-Aura:NewcolNo+Aura
```

```
                if Ac_NodeNo ( round(R),round(C))> -1
                    NewcolNo= colNo;
                    NewrowNo= rowNo;
                end
            end
        end

         %%---Updating myosin node features----

Mc_NodeNo(round(Mc_Node(NodeID,ROW)),round(Mc_Node(NodeID,COL)))= -1;
  %% reset the NodeNo in order to create it again afresh

        Mc_Node(NodeID,ROW)= NewrowNo;
        Mc_Node(NodeID,COL)= NewcolNo;

        Mc_NodeNo(round(NewrowNo),round(NewcolNo))=NodeID;  % new
place has you

        Combo(round(NewrowNo),round(NewcolNo))=Mc; % refresh combo

        NodeCountPerPixel((round(NewrowNo)),(round(NewcolNo))) =
NodeCountPerPixel((round(NewrowNo)),(round(NewcolNo))) + 1;
        NodeCountPerPixel((round(rowNo)), (round(colNo))) =
NodeCountPerPixel((round(rowNo)),(round(colNo))) - 1 ;
```

if trying to reach a staurated point then Increase myosin

```
         if noOfNodesAtNewPosition>5 %%increasing myosin when density
per pixel increases 5

         Mc_Node(NodeID,MYO_CONC)=0.6;

         end

    end
```

Published with MATLAB® R2020b
